# Effect of MPG Gene rs2858056 Polymorphism, Copy Number Variation, and Level of Serum MPG Protein on the Risk for Rheumatoid Arthritis

**DOI:** 10.1371/journal.pone.0120699

**Published:** 2015-03-10

**Authors:** Chung-Ming Huang, Shih-Yin Chen, Po-Hao Huang, Fuu-Jen Tsai

**Affiliations:** 1 Division of Immunology and Rheumatology, Department of Internal Medicine, China Medical University Hospital, Taichung, Taiwan; 2 Genetic Center, Department of Medical Research, China Medical University Hospital, Taichung, Taiwan; 3 Department of Pediatrics, China Medical University Hospital, Taichung, Taiwan; 4 Graduate Institute of Integrated Medicine, College of Chinese Medicine, China Medical University, Taichung, Taiwan; 5 Graduate Institute of Chinese Medical Science, College of Chinese Medicine, China Medical University, Taichung, Taiwan; University of Saarland Medical School, GERMANY

## Abstract

**Objective:**

This study examined the role of SNP rs2858056 of the MPG gene on the incidence and severity of rheumatoid arthritis (RA).

**Methods:**

This cohort study enrolled 365 RA patients and 375 age- and gender-matched healthy controls, all of whom had Han Chinese ethnicity and were from Taiwan. Gene polymorphism of the SNP rs2858056 of MPG was determined from genomic DNA. Allelic frequencies and genotypes were compared among cases and controls. Quantitation of rs2858056 copy number variation (CNV) was determined. Serum samples from RA patients and controls were analyzed to determine serum levels of MPG. The relationship between rs2858056 polymorphism and clinical manifestations of RA was evaluated.

**Results:**

Our results indicated a statistically significant difference in genotype frequency distributions at rs2858056 for RA patients and controls (p = 0.05) and a significant difference in allelic frequency in patients and controls (p = 0.04). Furthermore, there was a significantly greater level of serum MPG protein in patients than controls (p < 0.001). However, the cases and controls had no significant differences in MPG CNV (p = 0.12). We also did not detect any association of the MPG rs2858056 with rheumatoid factor (RF), extraarticular involvement, or bone erosion in the RA patients.

**Conclusion:**

Our study suggests that RA is associated with a polymorphism in the MPG gene (rs2858056) and increased serum level of the MPG protein.

## Introduction

Rheumatoid arthritis (RA) is an autoimmune disease characterized by joint inflammation and numerous peripheral inflammatory manifestations [1]. Patients experience chronic synovitis, destruction of joint tissue (cartilage and bone), and impaired joint function [2]. There are heritable risk factors [3–4], but the genetic basis of RA is not well understood. Previous studies identified nearly 60 genetic loci associated with susceptibility to RA in multiple populations. These include HLA-DRB1 [5], PTPN22 (encoding tyrosin-protein phosphotase non receptor type 22) [6], PADI4 (protein-arginine deiminase type 4) [7], TNFAIP3 (TNF-induced protein 3) [8], and CTLA-4 (cytotoxic T-lymphocyte protein4) [9].

Disruption of DNA repair appears increase the susceptibility to RA. DNA repair enzymes modulate the DNA damage from free-radicals, by repairing DNA mainly through four pathways: mismatch repair (MMR), nucleotide excision repair (NER), direct reversal, and base excision repair (BER). The oxidized base 7,8-dihydroxy-8-oxoguanine (8-oxo-G) is a mutagenic product of oxidative DNA damage and the BER pathway can excise misincorporated 8-oxo-G. In particular, N-methylpurine DNA glycosylase (*MPG*; MIM 156565) removes diverse damaged bases, including cytotoxic and mutagenic alkylation adducts of purine [10]. Previous studies have examined the role of the *MPG* gene in breast cancer and lung cancer [11–13]. Recently, Shao et al. reported that the DNA repair system was involved in the pathology of RA [14]. In particular, the T cells of RA patients failed to produce sufficient amounts of the DNA repair kinase, ataxia telangiectasia mutated (ATM).

Our previous study [15] investigated the association of four gene polymorphisms (rs3176364, rs710079, rs2858056, and rs2541632) with susceptibility to RA in 384 Taiwanese individuals (192 RA patients and 192 controls). The results showed that rs710079 and rs2858056 polymorphisms and the GCGC haplotype were associated with increased risk of RA progression. In the present study, we sought to confirm the role of MPG rs2858056 gene polymorphism, copy number variation (CNV), and level of serum MPG protein with the risk of RA. Thus, we compared the genotype and allele frequencies of the MPG polymorphism rs2858056, CNV, and serum MPG levels in RA patients and healthy individuals of Han Chinese ethnicity from central Taiwan. Furthermore, the relationship between rs2858056 polymorphism and clinical manifestations of RA was evaluated.

## Materials and Methods

### Patient selection

We enrolled 365 patients (285 women and 80 men) diagnosed with RA, based on the 1987 revised criteria of the American College of Rheumatology [16], and 375 unrelated healthy individuals (291 women and 84 men) from the general population were selected from health examination. The cases and controls were matched for age and sex, were of Han Chinese ethnicity, and lived in central Taiwan. The age of the RA patients was 22–78 (mean 51.1 ± 5.9) years. The age of the volunteers was 20–75 (mean 49.3 ± 5.1) years. The duration of disease in RA patients was 0–19 (mean 5.6 ± 1.4) years. The baselines characteristic of 365 patients with RA were shown in Table 1. Nephelometry was used to measure the rheumatoid factor (RF), and values of 20 IU/mL or more were considered positive. The presence or history of extra-articular manifestations ((1) subcutaneous rheumatoid nodules; (2) cutaneous Vasculitis; (3) eosinophilia; (4) lymphadenopathy; (5) pulmonary disease (pleurisy, interstitial fibrosis, nodular lung or pulmonary hypertension); (6) cardiac disease (pericarditis or conduction defect); (7) noncompressive neuropathy; (8) Raynaud’s phenomenon; or (9) sicca syndrome.) was recorded for all RA patients [17]. Radiographs of hands, wrists, and feet of patients were taken, and joint erosion was evaluated by a rheumatologist and a radiologist. We noted only the presence or absence of erosion, with no calculation of radiological score. To assess inflammatory activity we measured C-reactive protein (CRP), and erythrocyte sedimentation rate (ESR). All samples for genomic DNA isolation were collected by venipuncture. This study was approved by the Institutional Review Board (IRB) of China Medical University Hospital (Taichung, Taiwan) prior to patient enrollment. All participants provided written informed consent.

**Table 1:** Demographics and characteristics of MPG genetic polymorphisms in RA patients.

		MPG-3644	(rs2858056)		P value
	GG(n = 53)	CG(n = 135)	CC(n = 177)	total(n = 365)	
Age. Years (SD)*	51.2 (6.1)	49.7 (5.2)	52.3 (6.5)	51.1 (5.9)	0.67
Sex, female, n (%)	42 (79.2)	108 (80.0)	135 (76.3)	285 (78.1)	0.71
Disease duration, years,					
median (IQR) *	6 (8)	5 (7)	6 (8)	6 (8)	0.43
ESR**, mm/h (SD) *	35 (7.6)	28 (5.3)	32 (6.4)	31 (6.2)	0.72
CRP**, mg/dl (SD) *	1.7 (0.6)	1.5 (0.4)	1.5 (0.4)	1.5 (0.4)	0.93
Medications					
Hydroxylchroloqine, n (%)	42 (79.2)	105 (77.8)	137 (77.4)	284 (77.8)	0.96
Sulfasalazin, n (%)	29 (54.7)	67 (49.6)	92 (51.9)	187 (51.2)	0.81
Methotrexate, n (%)	43 (81.1)	110 (81.5)	142 (80.2)	295 (80.8)	0.96
Leflunomide, n(%)	21 (39.6)	62 (45.9)	70 (39.5)	153 (41.9)	0.49
NSAID**, n (%)	46 (86.8)	121 (89.6)	156 (88.1)	323 (88.5)	0.85
Steroid, n (%)	27 (50.9)	75 (55.6)	95 (53.7)	197 (53.9)	0.84
Anti-TNF**, n (%)	8 (15.1)	12 (8.9)	15 (8.5)	35 (9.6)	0.34

* Result are expressed as percentages, median (interquartile range, IQR), or mean (SD) as approciate.

** ESR, erythrocyte sedimentation rate; CRP, C-reacive protein; NSAID, non-steroidal anti-inflammatory drug; TNF, tumor necrosis factor

### SNP selection

*MPG* SNPs genotypes information was downloaded in December 2008 from the HapMap CHB + JPT population. HapMap genotypes were analyzed within Haploview and Tag SNPs were selected using the Tagger function by applying the following additional criteria: (i) a threshold minor allele frequency (MAF) in the HapMap CHB + JPT population of 0.05 for”tag SNPs”; and (ii) probe/primers that pass the qualification as recommended by the manufacturer (Applied Biosystems), to ensure a high genotyping success rate.

### Genomic DNA extraction and genotyping (polymerase chain reaction and restriction enzyme analysis)

Genomic DNA was prepared from peripheral blood according to the standard protocols of the DNA extraction kit (Genomic DNA kit, Roche, USA). Polymerase chain reaction (PCR) was used to identify the MPG rs2858056 polymorphism. Polymerase chain reaction was carried out in a total volume of 50 μL, containing 50 ng of genomic DNA, 2–6 pmol of each primer, 1× Taq polymerase buffer (1.5 mM MgCl_2_) and 0.5 units of AmpliTaq DNA polymerase (Perkin Elmer, Foster City, CA, USA). In the study of the EGFR rs2227983 SNP, the primers used were forward-5'-AGAGCTGAGATCACGCCATT-3' and reverse-5'-AGGGCATCCACTAGGAGGTT-3'. PCR amplification was performed in a PCR thermal cycler (GeneAmp PCR System 2400, Perkin Elmer). The PCR cycling conditions were as follows: one cycle at 95°C for 5 min, 35 cycles at 95°C for 30 seconds, 60°C for 30 seconds, 72°C for 45 seconds. One final cycle of extension at 72°C for 7 min, then holding at 25°C. The MPG rs2858056 SNP was analyzed by PCR amplification followed by restriction enzyme analysis with Hae III. Four fragments of 29 bp, 112 bp, 86 bp and 101 bp were present if the product was excised (CC homozygote). The uncut band showed up as three fragments of 29 bp, 169bp and 130 bp length on the gel. The reaction was then incubated for overnight at 65°C, and then 10μl of the products were loaded into a 3% agarose gel containing ethidium bromide for electrophoresis. The MPG rs2858056 SNP was categorized as excisable (CC homozygote), non-excisable (CG homozygote), and (GG heterozygote).

### Quantitation of MPG copy number

A pre-designed TaqMan Copy Number assay (Assay ID Hs00453520_cn, Applied Biosystems, USA) was used for quantitation of MPG copy number. PCR reactions contained 2× TaqMan PCR Master Mix (Applied Biosystems, USA), 20× TaqMan Copy Number Assay (Applied Biosystems, USA), 20× TaqMan Copy Number Reference Assay-RNase P-Human (Cat No. 4403328, Applied Biosystems, USA), and 10 ng of genomic DNA. The TaqMan RNase P control reagents kit (Applied Biosystems, USA) was used as an endogenous control. After initial denaturation for 10 min at 95°C, 40 cycles were run, each consisting of denaturation (95°C for 15 s), and annealing (60°C for 60 s). The fluorescence signal was measured using the ABI Prism 7900 Real Time PCR System.

### Quantitative determination of MPG in serum

Serum samples were diluted with buffer so they were within the range of the standard curve, and were then quantified by an enzyme-linked immunosorbent assay (ELISA, E97625Hu, Uscn Life Science Inc, Wuhan, China). The serum MPG was bound to monoclonal mouse antibodies against human MPG, which were immobilized on the surface of microtiter plates. After washing, bound MPG was quantified by adding a rabbit anti-human MPG antibody. The bound rabbit antibody was measured by a peroxidase-labeled goat anti-rabbit antibody. The amount of converted substrate was directly proportional to the amount of bound human MPG, and was determined by measurement of OD_450 nm_.

### Statistical analysis

The genotype and allele frequencies of MPG SNP rs2858056 in RA patients and controls were compared using the chi-square test. Allelic frequency was expressed as a percentage of the total number of alleles. Differences between genotypes and allele frequencies and deviations from the Hardy-Weinberg equilibrium were analyzed using the χ2 test and Fisher’s exact test, depending on the minimum expected values. A *p-*value less than 0.05 was considered statistically significant. Odds ratios (ORs) and 95% confidence intervals (95% CIs) were calculated for allelic frequency of MPG SNP rs2858056 and for CNV. The Mann-Whitney U test or the Kruskal-Wallis test was used for non-parametric comparisons, and Student’s *t-*test or one-way analysis of variance was used for parametric comparisons. All statistical analysis was performed with SPSS version 11.

## Results

### Genotype and allele frequency distributions of MPG rs2858056 SNP

Table 2 shows the frequencies of the genotype MPG rs2858056 in the RA and control groups. There was a significant difference in the distribution of the MPG rs2858056 gene polymorphism between patients and controls (OR for GG genotype: 1.79, 95% CI: 1.11–2.89, *p* = 0.05). In addition, there was a significant difference in allele frequency of MPG rs2858056 between patients and controls (OR for G allele: 1.26, 95% CI: 1.01–1.57, *p* = 0.04). The statistic power with alpha at 0.05 was more than 89% in this study (89.7%).

**Table 2:** Genotype and allele frequencies of MPG genetic polymorphisms in RA patients (n = 365) and controls (n = 375).

	Patients, n (%)	Controls, n (%)	OR (95% CI)	*P* value
**MPG-3644 (rs2858056)**				
CC	177 (48.5)	197 (52.5)	Ref	0.05
CG	135 (37.0)	145 (38.7)	1.04 (0.76–1.41)	
GG	53 (14.5)	33 (8.8)	1.79 (1.11–2.89)	
**Allele frequency**				
Allele C	489 (67.0)	539 (71.9)	Ref	0.04
Allele G	241 (33.0)	211 (28.1)	1.26 (1.01–1.57)	

### Abnormal MPG CNV in RA patients

Blood leukocyte gDNA samples were available from 235 RA patients (64.4%) and 223 healthy controls (59.5%); for the other subjects, insufficient gDNA was available for quantification of MPG CNV. The presence of RA was not associated with abnormal MPG CNV (Table 3, *p* = 0.12, Fisher’s test). Most patients and controls had 2 copies of the MPG gene (100% of controls and 98.3% of patients). However, 4 RA patients (1.7%) had 3 copies of the MPG gene, and none of the healthy controls (0%) had 3 copies.

**Table 3:** Distribution of MPG copy number variation (CNV) in RA patients (n = 235) and controls (n = 223).

	Controls, n (%)	Patients, n (%)	*P* value(Chi-square test)	*P* value(Fisher's test)
**MPG copies**				
2	223 (100)	231 (98.3)	0.05	0.12
3	0 (0)	4 (1.7)		

### Increased serum level of MPG in RA patients

**Fig 1:**
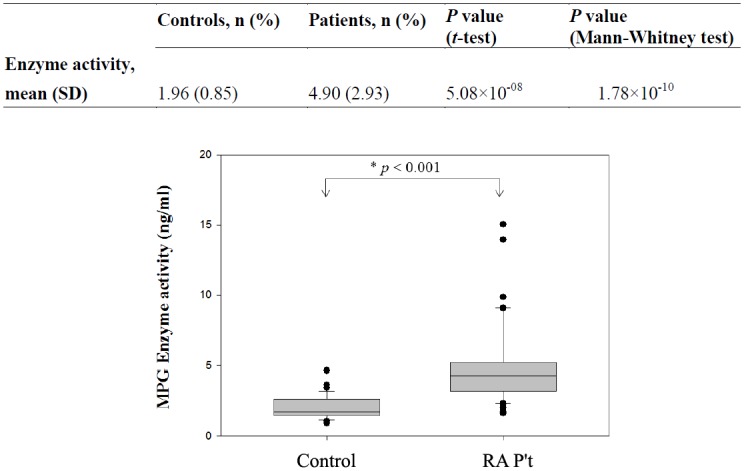
Serum level of MPG protein in RA patients (n = 40) and controls (n = 39).

**Fig 2:**
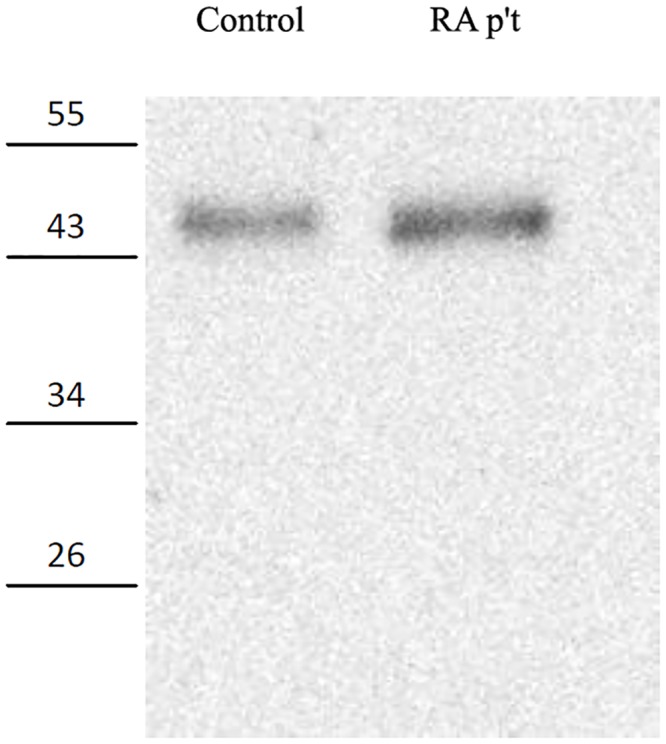
Representative MPG western blot assays of an RA patient and a control patient.

Serum levels of MPG were available from 40 RA patients (10.7%) and 39 healthy controls (10.4%); for the other subjects, insufficient serum was available for quantification of serum MPG (Fig. 1). The results indicate a significantly higher level of MPG in patients than in controls (4.90 ± 2.93 ng/mL *vs*. 1.96 ± 0.85 ng/mL, *p* < 0.001). This was confirmed by western blot assays for MPG (Fig. 2).

### Clinical signs and findings and genotype of MPG polymorphisms in RA patients

Clinical manifestations and laboratory findings of the RA patients are shown in Table 4. The associations of the genotype MPG rs2858056 with particular clinical features of RA were examined in the 365 Chinese patients. We did not detect any association of the MPG rs2858056 genotype with the RF or extra-articular involvement in the RA patients (P = 0.90 and P = 0.18, respectively). We observed increased frequencies of bone erosion among the RA patients with GG genotype (54.7%) when compared with CC genotype (37.9%). However, this difference did not reach statistical significance (P = 0.07, χ2 = 5.33).

**Table 4:** Relationships between MPG-3644 (rs2858056) genotype and clinical signs and findings in patients with RA.

	GG(n = 53)	CG(n = 135)	CC(n = 177)	total(n = 365)	*P* value
**Rheumatoid factor (+)**	38(71.7%)	101(74.8%)	130(73.4%)	269(73.7%)	0.90
**Extra-articular involvement**	21(39.6%)	65 (48.1%)	67 (37.9%)	153(41.9%)	0.18
**Bone erosion**	29(54.7%)	62 (45.9%)	67 (37.9%)	156(42.7%)	0.07

## Discussion

Inflammation results from a variety of cellular signals and the formation of reactive oxygen species (ROS), which threaten the integrity of cellular DNA. The oxidized base 7,8-dihydroxy-8-oxoguanine (8-oxo-G) is a mutagenic product of oxidative DNA damage. However, misincorporated 8-oxo-G can be excised by the base excision repair (BER) pathway, and many DNA repair enzymes, including MUTYH, OGG1, MPG, TDG, UNG, EGFR, and SMUG1, are part of, or related to, this pathway [10].

Previous studies have examined the roles of genes related to DNA repair in various cancers, including lung cancer, breast cancer [11–13], gastric cancer [17], head and neck squamous cell carcinomas (HNSCC) [18], and colorectal cancer (CRC) [19]. In addition, there is evidence that ROS-induced DNA damage may contribute to tumor development in a murine model of chronic inflammation [20]. These studies led to our examination of genetic and allelic variations of enzymes that repair ROS-induced DNA damage and the risk of RA.

Our previous research examined the gene and allele frequencies of polymorphisms in DNA repair-related genes (including OGG1, MPG, UNG, and EGFR), and compared the haplotypes of RA patients and healthy controls in a Taiwan population [15, 21–23]. In particular, our 2010 study [15] investigated that the association of four MPG polymorphisms (rs3176364, rs710079, rs2858056, and rs2541632) with RA by examination of 192 patients and 192 healthy controls. The results indicated statistically significant differences in genotype frequency distributions of rs710079 and rs2858056 between RA patients and controls (*p* = 0.04 and *p* = 0.029, respectively). In the present study, we increased the case numbers (365 RA patients, 375 healthy controls) to increase the statistical power of the analysis and focused on the association of the MPG rs2858056 polymorphism with the risk of RA. The results showed that there was a statistically significant difference in genotype and allele frequency distributions between RA patients and healthy controls. In particular, individuals with the G genotype at rs2858056 have increased risk for RA. We also used Hardy—Weinberg equilibrium (HWE) testing for data quality control. The results showed that the control group of rs2858056 SNP is under the null hypothesis of no departure from HWE. In the data set, the p value under 0.05 is obtained in RA patients group of rs2858056 SNP, giving the evidence of departure from HWE. It shown that rs2858056 SNP maybe plays an important role in RA development.

We also evaluated the relationship of RA with MPG CNV (Table 2) and MPG expression (Fig. 1). Several large-scale whole-genome studies and focused studies have documented widespread CNV in the human genome [24–27]. This variation accounts for roughly 12% of human genomic DNA, and each variation ranges from about one kilobase to several megabases in size [28]. Gene copy number is often altered in the presence of systemic disease. For instance, the EGFR copy number is elevated in non-small cell lung cancer [29], an elevated copy number of CCL3L1 is associated with lower susceptibility to HIV infection [30], and a low copy number of FCGR3B (the CD16 cell-surface immunoglobulin receptor) increases the susceptibility to systemic lupus erythematosus and similar inflammatory autoimmune disorders [27]. However, although CNV has been proposed to explain the elusive hereditary causes of complex diseases, such as rheumatoid arthritis, the most common CNVs have little or no role in causing disease [31]. Similarly, the results of the present study indicate that RA patients did not have abnormal MPG CNV. Most patients and controls had 2 copies of the MPG gene (100% of controls, 98.3% of patients). However, 4 of the patients (1.7%) had 3 copies, but none of the healthy controls had 3 copies.

We collected blood samples from patients and controls and determined serum MPG levels to verify the functional expression of MPG rs2858056 SNP. The results indicated significantly higher levels of serum MPG protein in patients than controls. In agreement, our research on the role of EGFR gene rs17337023 polymorphism, copy number variation, and serum levels in RA indicated no effect of CNV of this gene (unpublished observation).

The etiology of RA is still incompletely characterized, and this is a very active area of research worldwide. The present investigation examined whether an MPG SNP is associated with RA susceptibility. To our knowledge, from a search of reports of the literature on MEDLINE, an association between the gene polymorphism of MPG rs2858056 and RA has not been demonstrated before. In the present study, the significant differences were observed in the allelic frequencies and genotype of MPG rs2858056 gene polymorphism between RA patients and healthy controls. However, we did not find any relationships between MPG rs2858056 and clinical manifestations and laboratory profiles (RF and extra-articular involvement) of RA in the Chinese patients. Although we observed increased frequencies of bone erosion among RA patients with the GG genotype (54.7%) when compared with CC genotype (37.9%), this difference did not reach statistical significance (P = 0.07). A much larger study group will be required to verify the relationship between disease phenotypes and genotypes.

The generalizability of our results is limited because all of the patients were from single center in Taiwan. Nonetheless, our results strongly suggest a significant role of MPG rs2858056 gene polymorphisms in the risk for RA among subjects from Taiwan. The identification of MPG alterations as a risk factor for RA in Taiwan requires further clinical testing worldwide, especially in populations with different ethnicities.

In conclusion, the present study showed a correlation between increased MPG expression and MPG gene polymorphism with RA.
